# Association of change in alcohol consumption with cardiovascular disease and mortality among initial nondrinkers

**DOI:** 10.1038/s41598-020-70304-7

**Published:** 2020-08-07

**Authors:** Jun Young Chang, Seulggie Choi, Sang Min Park

**Affiliations:** 1 Department of Neurology, Asan Medical Center, Seoul, Republic of Korea; 2 Department of Biomedical Sciences, Seoul National University Graduate School, Seoul National University College of Medicine, 101 Daehangno, Jongno-gu, Seoul, 110-744 Korea

**Keywords:** Cardiology, Diseases, Health care, Medical research, Neurology, Risk factors

## Abstract

There is a paucity of studies on the influence of alcohol intake among non-drinkers. We evaluated the association between an increase in alcohol consumption and primary prevention of major adverse cardiovascular events (MACE) among non-drinkers. Data collected by the National Health Insurance Service in the Korea between 2007 and 2013 were analysed. A total of 112,403 subjects were included and followed up from 1 January 2011 to 31 December 2013. Increases in alcohol consumption, measured as glasses per day, at the second medical check-up, were categorized into maintenance of nondrinking (0), > 0– ≤ 1, > 1– ≤ 2, > 2– ≤ 4, and > 4. Hazard ratios (HRs) for MACE and all-cause mortality on increase in alcohol consumption were calculated. Compared to that in non-drinkers at the second check-up, the risk of MACE significantly decreased among the subjects with an increase in alcohol consumption to ≤ 1 glass per day (HR 0.79, 95% CI 0.68–0.92). However, a light increase in alcohol consumption did not reduce the risk of stroke or all-cause mortality (stroke, HR 0.83, 95% CI 0.68–1.02; all-cause mortality, HR 0.89, 95% CI 0.73–1.09). Compared to continual non-drinkers, those who drank > 2 glass per day had higher risk for death due to external causes (aHR 2.06, 95% CI 1.09–3.90). The beneficial effect of light increments in alcohol consumption on the occurrence of MACE may have resulted from the inappropriate inclusion of sick quitters, who maintained a nondrinking status, in the reference group.

## Introduction

Many epidemiologic studies have investigated the influence of alcohol consumption on increment in cardiovascular events, including stroke, which showed J-shaped association. Heavy drinking increases the risk of stroke, myocardial infarction, atrial fibrillation, and congestive heart failure by rebound elevation of platelet aggregation and blood pressure^1–4^. Light-to-moderate alcohol intake may have preventive effects on cardiovascular disease by increasing high-density lipoprotein and decreasing platelet aggregation and fibrinogen concentration^5,6^. A low level of alcohol intake decreases incidence of diabetes by enhancing insulin sensitivity^7^. Despite the beneficial influence of light-to-moderate alcohol consumption, non-drinkers are not advised to start drinking according to the guidelines from primary prevention of stroke^8^ The results of a recent study showed that no level of alcohol consumption is associated with health benefit, as the net benefit from low-dose alcohol consumption on cardiovascular disease prevention and diabetes was attenuated by its harmful effect of an increase in cancer or other injuries ^9^.

There is a paucity of studies on the influence of alcohol intake among non-drinkers. Further, prior studies on alcohol intake have had several methodological problems, which make their conclusions unreliable. The purpose of our study was to evaluate whether an increase in alcohol consumption is still beneficial in primary prevention of major adverse cardiovascular events (MACE), including stroke, coronary artery disease, or cardiovascular death, and all-cause mortality among initial non-drinkers.

## Results

Among the 490,255 subjects in the entire study sample, including the drinkers who underwent both the first (2007–2008) and second (2009–2010) medical checkups, 161,383 (32.9%) were initial nondrinkers at the first health examination. After excluding the subjects with cardiovascular events before the index date (43,632), death before the index date (255), missing information on alcohol intake (3,187), and missing covariate information (1906), 112,403 subjects (22.9%) were finally included in the present analysis (Supplementary Fig. 1). Of the non-drinkers enrolled, 96,716 (86.0%) maintained their non-drinking status at the second medical check-up; 10,621 (9.4%) had begun to drink > 0– ≤ 1 glass per day; 2,476 (3.6%), > 1– ≤ 2 glasses per day; 1784 (1.6%), > 2– ≤ 4 glasses per day; and 806 (0.7%), > 4 glasses per day. The characteristics of the subjects who maintained their non-drinking status at the second check-up are presented in Table 1. The mean age was 59.0 (± 8.6) years, and 70% were female. The proportions of subjects with a non-smoking status and no physical activity were 83.9% and 53.3%, respectively. The proportion of subjects with Charlson’s comorbidity index (CCI) ≥ 3 was 25.7%. Compared with the subjects who maintained a nondrinking status, those who started drinking were more likely to be male, in the highest quartile of household income, a past or current smoker, physically active, and to have fewer comorbidities (Supplementary Table 1). Systolic blood pressure and the fasting serum glucose level tend to increase and the total cholesterol level tends to decrease as the alcohol consumption dose increases (Table 1).

**Figure 1:**
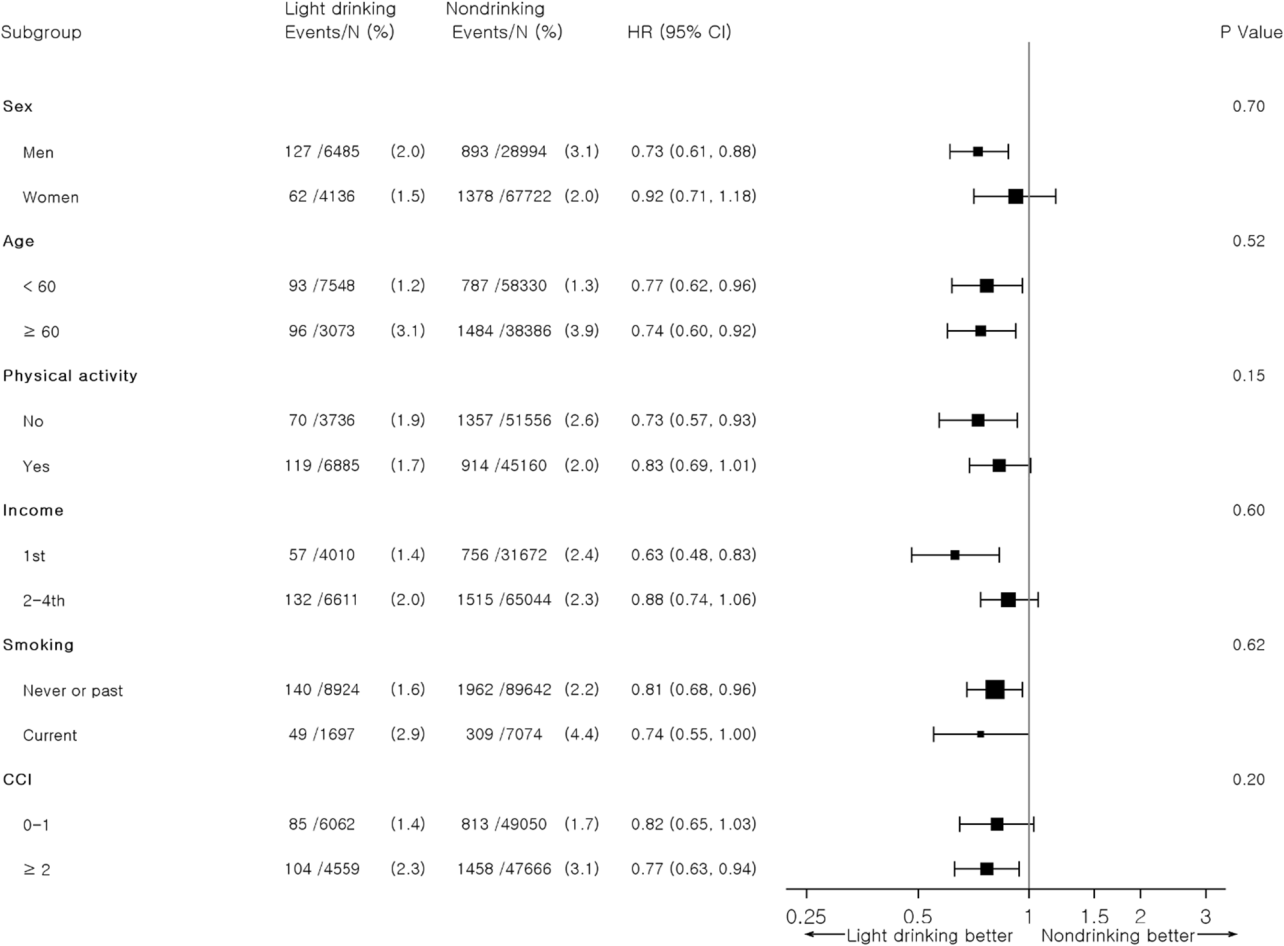
Subgroup analysis of the association of alcohol consumption change with major adverse cardiovascular events among initial non-drinkers.

**Table 1:** Descriptive characteristics of the study population.

	Alcohol intake during second health examination (drinks per day)					
	Total	0	> 0– ≤ 1	> 1– ≤ 2	> 2– ≤ 4	> 4
Number of participants (%)	112,403	96,716 (86.0)	10,621 (9.4)	2,476 (3.6)	1,784 (1.6)	806 (0.7)
Age, years, mean (SD)	58.7 (8.5)	59.0 (8.6)	56.8 (7.8)	56.6 (7.6)	56.3 (7.3)	55.1 (6.6)
Sex, N (%)						
Men	40,035 (35.6)	28,994 (30.0)	6,485 (61.1)	2,132 (86.1)	1,639 (92.4)	775 (96.2)
Women	72,368 (64.4)	67,722 (70.0)	4,136 (38.9)	344 (13.9)	135 (7.6)	31 (3.9)
Household income, quartiles, N (%)						
1st (highest)	37,640 (33.5)	31,672 (32.8)	4,010 (37.8)	958 (38.7)	685 (38.4)	315 (39.1)
2nd	32,879 (29.3)	28,277 (29.2)	3,047 (28.7)	761 (30.7)	545 (30.6)	249 (30.9)
3rd	24,482 (21.8)	21,356 (22.1)	2,153 (20.3)	475 (19.2)	338 (19.0)	160 (19.9)
4th (lowest)	17,402 (15.5)	15,411 (15.9)	1,411 (13.3)	282 (11.4)	282 (11.4)	82 (10.2)
Smoking, N (%)						
Never smoker	89,331 (79.5)	81,139 (83.9)	6,505 (61.3)	965 (39.0)	519 (29.1)	203 (25.2)
Past smoker	12,538 (11.2)	8,503 (8.8)	2,419 (22.8)	758 (30.6)	603 (33.8)	255 (31.6)
Current smoker	10,534 (9.4)	7,074 (7.3)	1,697 (16.0)	753 (30.4)	662 (37.1)	348 (43.2)
Physical activity, times per week, N (%)						
0	57,150 (50.8)	51,556 (53.3)	3,736 (35.2)	902 (36.4)	653 (36.6)	303 (37.6)
1–2	32,126 (28.6)	26,032 (26.9)	4,242 (39.9)	896 (36.2)	664 (37.2)	292 (36.2)
3–4	16,690 (14.9)	13,782 (14.3)	1,910 (18.0)	490 (19.8)	346 (19.4)	162 (20.1)
≥ 5	6,437 (5.7)	5,346 (5.5)	733 (6.9)	188 (7.6)	121 (6.8)	49 (6.1)
Body mass index, kg/m^2^, mean (SD)	23.7 (2.9)	23.7 (2.9)	23.8 (2.7)	24.1 (2.8)	24.3 (2.8)	24.6 (2.8)
Systolic blood pressure, mmHg, mean (SD)	123.3 (15.1)	123.2 (15.2)	123.0 (14.5)	125.7 (14.8)	127.0 (14.8)	127.4 (14.8)
Fasting serum glucose, mg/dL, mean (SD)	98.3 (22.6)	98.0 (22.3)	98.8 (22.6)	102.1 (26.3)	104.2 (26.3)	106.0 (28.6)
Total cholesterol, mg/dL, mean (SD)	202.5 (37.1)	202.8 (37.2)	201.0 (36.2)	199.9 (36.4)	198.5 (35.7)	198.7 (36.6)
Charlson comorbidity index, N (%)						
0	24,261 (21.6)	20,096 (20.8)	2,735 (25.8)	671 (27.1)	523 (29.3)	236 (29.3)
1	33,927 (30.2)	28,954 (29.9)	3,327 (31.3)	812 (32.8)	567 (31.8)	267 (33.1)
2	26,300 (23.4)	22,840 (23.6)	2,416 (22.8)	530 (21.4)	345 (19.3)	169 (21.0)
≥ 3	27,915 (24.8)	24,826 (25.7)	2,143 (20.2)	463 (18.7)	349 (19.6)	134 (16.6)

Acronyms: *SD* standard deviation.

During the follow-up period from 1 January 2011 to 31 December 2013, a total of 2,271 individuals experienced MACE, 954 individuals experienced coronary artery disease, and 1,332 individuals experienced stroke during a 285,301 person-year follow-up. A total of 1,213 subjects died during the 288,297 person-year follow-up. Compared with the group that maintained their non-drinking status at the second check-up, subjects with an increase in alcohol consumption to > 0– ≤ 1 glass per day (adjusted HR 0.79, 95% CI 0.68–0.92) showed a significant decrease in MACE risk. The adjusted HRs (95% CI) were 0.85 (0.64–1.11) for an increase in alcohol consumption to > 1– ≤ 2 glasses; 0.95 (0.71–1.28), for > 2– ≤ 4 glasses; and 1.05 (0.69–1.61), for > 4 glasses, compared to that in the group maintaining a nondrinking state. An increase in alcohol consumption to > 0 to ≤ 1 glasses per day also reduced the risk of coronary artery disease (HR 0.75, 95% CI 0.60–0.93) but not stroke, all-cause mortality, or death due to external causes (stroke, HR 0.83, 95% CI 0.68–1.02; all-cause mortality, HR 0.89, 95% CI 0.73–1.09; Table 2). Compared to continual non-drinkers, those who drank > 2 drinks per day of alcohol had higher risk for death due to external causes (aHR 2.06, 95% CI 1.09–3.90).

**Table 2:** Hazard ratios for cardiovascular disease, all-cause mortality, and death due to external causes according to change in alcohol consumption among initial non-drinkers.

	Alcohol intake during second health examination (drinks per day)				
	0	> 0– ≤ 1	> 1– ≤ 2	> 2– ≤ 4	> 4
MACE					
Events	2,271	189	55	46	22
Person-years	285,301	31,442	7,304	5,249	2,373
aHR (95% CI)	1.00 (reference)	0.79 (0.68–0.92)	0.85 (0.64–1.11)	0.95 (0.71–1.28)	1.05 (0.69–1.61)
Coronary artery disease					
Events	954	88	27	22	14
Person-years	285,301	31,442	7,304	5,249	2,373
aHR (95% CI)	1.00 (reference)	0.75 (0.60–0.93)	0.79 (0.53–1.16)	0.83 (0.54–1.28)	1.18 (0.69–2.02)
Stroke					
Events	1,332	102	29	24	8
Person-years	285,301	31,442	7,304	5,249	2,373
aHR (95% CI)	1.00 (reference)	0.83 (0.68–1.02)	0.93 (0.64–1.35)	1.07 (0.71–1.61)	0.85 (0.42–1.71)
All-cause mortality					
Events	1,213	108	25	25	10
Person-years	288,297	31,698	7,392	5,317	2,402
aHR (95% CI)	1.00 (reference)	0.89 (0.73–1.09)	0.76 (0.51–1.14)	1.02 (0.69–1.53)	1.11 (0.58–2.08)
Death due to external causes	0	> 0–≤ 2		> 2	
Events	157	21		11	
Person-years	288,297	288,297		39,090	
aHR (95% CI)	1.00 (reference)	0.89 (0.56–1.42)		2.06 (1.09–3.90)	

Adjusted hazard ratio calculated by Cox proportional hazards regression after adjustments for age, sex, household income, smoking, physical activity, body mass index, systolic blood pressure, fasting serum glucose, total cholesterol, and Charlson comorbidity index.

Death due to external causes, the exposure group were composed of 0, > 0– ≤ 2, and > 2 drinks per day due to the small number of events.

Acronyms: *MACE* major adverse cardiovascular events, *aHR* adjusted hazard ratio, *CI* confidence interval.

The associations of alcohol consumption change with MACE according to the sex, age, physical activity, income, smoking, and medical comorbidities have been listed in Table 3.

**Table 3:** Stratified analysis of the association of alcohol consumption change with major adverse cardiovascular events among initial non-drinkers according to subgroups of sex, age, physical activity, smoking, and Charlson comorbidity index.

	Alcohol intake during second health examination (drinks per day)					*p* for interaction
	0	> 0– ≤ 1	> 1– ≤ 2	> 2– ≤ 4	> 4	
Sex						
Men	1.00 (reference)	0.73 (0.61–0.88)	0.81 (0.61–1.09)	0.95 (0.70–1.29)	1.04 (0.68–1.60)	0.70
Women	1.00 (reference)	0.92 (0.71–1.18)	1.00 (0.45–2.23)	0.45 (0.06–3.21)	-	
Age, years						
< 60	1.00 (reference)	0.77 (0.62–0.96)	0.86 (0.60–1.24)	0.78 (0.51–1.19)	1.03 (0.62–1.70)	0.52
≥ 60	1.00 (reference)	0.74 (0.60–0.92)	0.72 (0.48–1.08)	0.94 (0.62–1.42)	0.70 (0.31–1.57)	
Physical activity						
No	1.00 (reference)	0.73 (0.57–0.93)	0.55 (0.33–0.92)	1.03 (0.67–1.60)	1.48 (0.85–2.57)	0.15
Yes	1.00 (reference)	0.83 (0.69–1.01)	1.05 (0.76–1.45)	0.88 (0.58–1.32)	0.73 (0.37–1.41)	
Income						
1st	1.00 (reference)	0.63 (0.48–0.83)	0.71 (0.43–1.15)	0.77 (0.45–1.32)	1.19 (0.61–2.32)	0.60
2–4th	1.00 (reference)	0.88 (0.74–1.06)	0.92 (0.66–1.28)	1.05 (0.74–1.50)	0.96 (0.55–1.66)	
Smoking						
Never or past smoker	1.00 (reference)	0.81 (0.68–0.96)	1.04 (0.75–1.44)	0.87 (0.56–1.34)	1.51 (0.89–2.57)	0.62
Current smoker	1.00 (reference)	0.74 (0.55–1.00)	0.60 (0.37–0.96)	0.94 (0.62–1.42)	0.60 (0.30–1.22)	
Charlson comorbidity index						
0–1	1.00 (reference)	0.82 (0.65–1.03)	0.77 (0.51–1.17)	1.24 (0.85–1.81)	1.36 (0.80–2.33)	0.20
≥ 2	1.00 (reference)	0.77 (0.63–0.94)	0.90 (0.63–1.30)	0.68 (0.42–1.10)	0.74 (0.37–1.49)	

Adjusted hazard ratio calculated by Cox proportional hazards regression after adjustments for age, sex, household income, smoking, physical activity, body mass index, systolic blood pressure, fasting serum glucose, total cholesterol, and Charlson comorbidity index.

There was no significant heterogeneity across sex, age, physical activity, smoking, and CCI score with regard to the protective effect of an increase in alcohol consumption to > 0– ≤ 1 glasses per day on MACE (Fig. 1).

**Figure 2:**
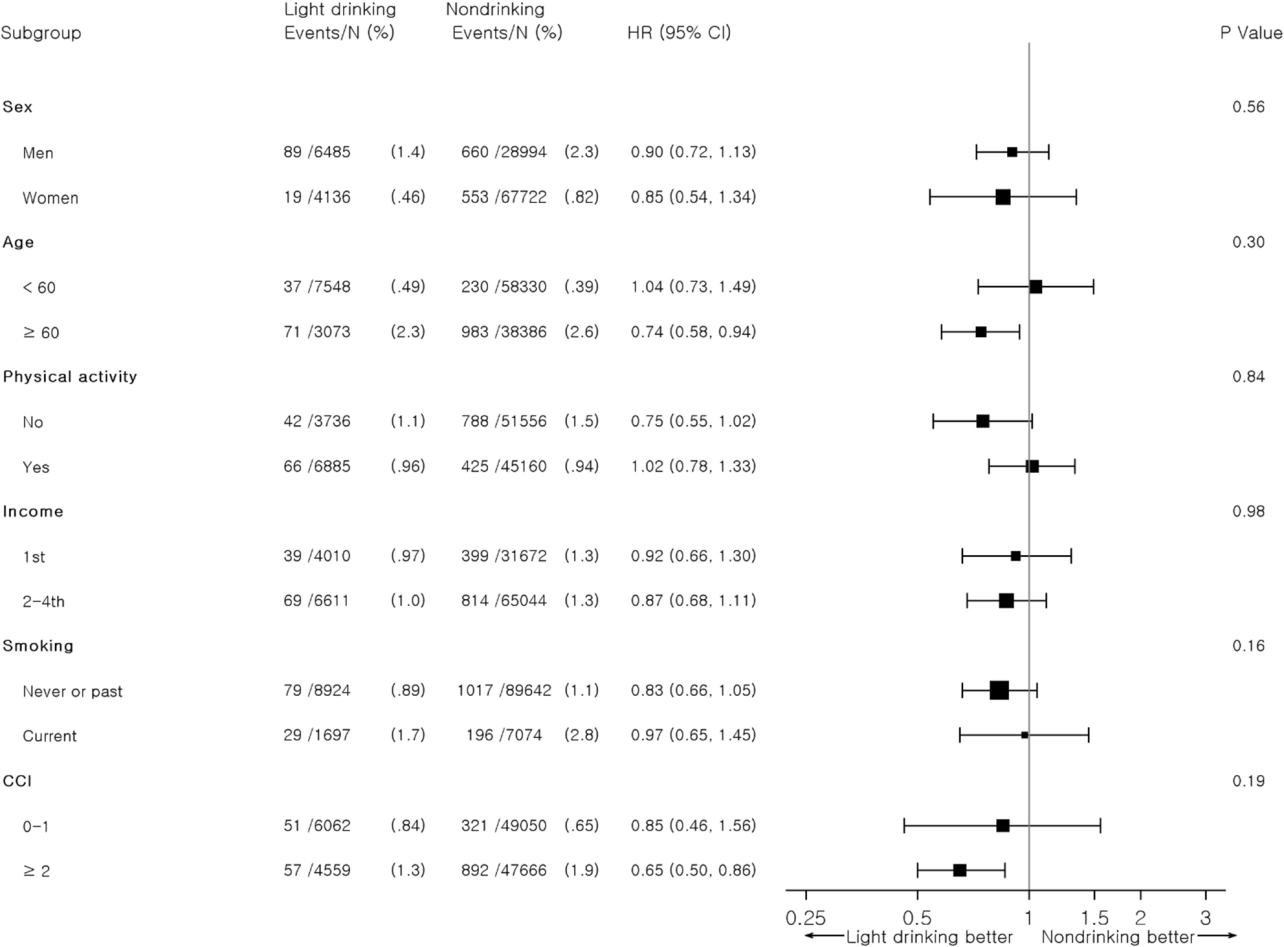
Subgroup analysis of the association of alcohol consumption change with all-cause mortality among initial non-drinkers.

There was no significant interaction between alcohol consumption change and all-cause mortality across the prespecified variables. The risk of all-cause mortality tended to be reduced by alcohol consumption to > 0 to ≤ 1 glasses per day in the subjects aged ≥ 60 years and whose CCI values were ≥ 2, though not statistically significant (Table 4 and Fig. 2).

**Table 4:** Stratified analysis of the association of alcohol consumption change with all-cause mortality among initial non-drinkers according to subgroups of sex, age, physical activity, smoking, and Charlson comorbidity index.

	Alcohol intake during second health examination (drinks per day)					*p* for interaction
	0	> 0– ≤ 1	> 1– ≤ 2	> 2– ≤ 4	> 4	
Sex						
Men	1.00 (reference)	0.90 (0.72–1.13)	0.81 (0.54–1.22)	0.96 (0.63–1.46)	1.13 (0.61–2.13)	0.56
Women	1.00 (reference)	0.85 (0.54–1.34)	–	3.48 (0.86–14.00)	–	
Age, years						
< 60	1.00 (reference)	1.04 (0.73–1.49)	0.53 (0.24–1.22)	1.05 (0.53–2.08)	0.21 (0.03–1.54)	0.30
≥ 60	1.00 (reference)	0.74 (0.58–0.94)	0.74 (0.47–1.17)	0.76 (0.40–1.25)	1.36 (0.71–2.64)	
Physical activity						
No	1.00 (reference)	0.75 (0.55–1.02)	0.84 (0.49–1.44)	1.23 (0.75–2.04)	1.19 (0.53–2.67)	0.84
Yes	1.00 (reference)	1.02 (0.78–1.33)	0.68 (0.37–1.24)	0.80 (0.41–1.56)	1.01 (0.37–2.73)	
Income						
1st	1.00 (reference)	0.92 (0.66–1.30)	0.91 (0.46–1.77)	0.98 (0.48–1.99)	0.84 (0.21–3.41)	0.98
2–4th	1.00 (reference)	0.87 (0.68–1.11)	0.71 (0.43–1.17)	1.05 (0.64–1.70)	1.17 (0.58–2.37)	
Smoking						
Never or past smoker	1.00 (reference)	0.83 (0.66–1.05)	0.24 (0.10–0.59)	1.01 (0.66–1.79)	0.79 (0.29–2.10)	0.16
Current smoker	1.00 (reference)	0.97 (0.65–1.45)	1.35 (0.85–2.16)	0.94 (0.48–1.85)	1.44 (0.63–3.27)	
Charlson comorbidity index						
0–1	1.00 (reference)	1.33 (0.98–1.80)	0.85 (0.46–1.56)	1.34 (0.72–2.47)	1.58 (0.65–3.85)	0.19
≥ 2	1.00 (reference)	0.66 (0.50–0.86)	0.65 (0.38–1.11)	0.85 (0.50–1.44)	0.80 (0.33–1.94)	

Adjusted hazard ratio calculated by Cox proportional hazards regression after adjustments for age, sex, household income, smoking, physical activity, body mass index, systolic blood pressure, fasting serum glucose, total cholesterol, and Charlson comorbidity index.

## Discussion

We demonstrated that a light increase in alcohol consumption to ≤ 1 glass per day from the initial non-drinker status significantly reduced the risk of MACE, but did not reduce the risk of all-cause mortality. To our knowledge, this is the first study to investigate the influence of an increase in alcohol consumption on cardiovascular disease occurrence and all-cause mortality among initial non-drinkers.

We evaluated the effect of a change in alcohol consumption within the same cohort at different time points. We designated a reference group that maintained their non-drinking status at the next health check-up. Further, we performed multivariable analysis using more comprehensive covariate adjustment, including adjustment for demographics (age, sex, BMI), socioeconomic status, health-related behaviour (smoking habit, physical activity), laboratory findings (systolic blood pressure and serum glucose and cholesterol levels), and medical comorbidities.

Low-to-moderate alcohol intake has been regarded as protective against ischemic heart disease and mortality^10,11^. However, more recent evidence has revealed there exists no threshold for the beneficial effect of alcohol^9,12,13^. Most epidemiologic studies compared risks of diseases with regard to an amount of alcohol intake and there are few studies on the effect of alcohol consumption change on disease occurrence among non-drinkers. The results of the current study demonstrated some protective effects on cardiovascular diseases with a light increase in alcohol consumption (> 0– ≤ 1 glass per day), regardless of age, sex, physical activity, social income, and comorbid illnesses, in initial non-drinkers. However, a light increase in alcohol consumption does not reduce all-cause mortality among non-drinkers. Even an increase in alcohol consumption to > 2 glasses per day significantly increased the risk of death due to external causes (HR 2.06, 95% CI 1.09–3.90). Due to the lack of evidence of the benefit, it would be an inappropriate recommendation to start light alcohol drinking among nondrinkers.

Alcohol consumption is an important risk factor for diseases, disability, and mortality^14^. The harmful effect of alcohol is multifactorial. Alcohol use disorders, including alcohol dependence and abuse, are the second most important cause of disabling disease and injury, especially among men. Alcohol is a component cause of many disease categories, including infectious diseases, neuropsychiatric illness, malignancy, cardiovascular diseases, hepatobiliary diseases, and injuries^15^. Despite many harmful effects of alcohol consumption, light-to-moderate alcohol ingestion has been considered to have health-protective effects especially against cardiovascular diseases and diabetes. Light alcohol consumption (30 g of ethanol per day) was associated with favourable change of lipids and hemostatic factors by increasing high density lipoprotein-cholesterol (HDL-C) and apolipoprotein A1, decreasing in fibrinogen and glycated hemoglobin levels^16,17^. However, the increased global burden of disease by alcohol consumption far outweighed the slight benefit of cardiovascular disease protection from light alcohol consumption^9^. The results of the current study show that the association is not very different among initial non-drinkers.

Recent evidence has revealed that the J-shaped association between alcohol consumption and mortality can be attributed to many biases, including misclassification of the heterogeneous non-drinking group in a single category, lack of adjustment by unmeasured confounders, and the sick quitter phenomenon^18–21^. The results from high-quality studies free from abstainer bias showed a linear dose–response relationship between alcohol consumption and mortality^13^. The beneficial effect of light alcohol consumption reported in prior studies may result from inappropriate selection of the reference group, which includes formal drinkers who quit drinking before study enrolment. The proportion of participants having potentially adverse confounders was higher among non-drinkers than among moderate drinkers^20^. This group of patients is also known to have higher risk of depression and mortality.

The observed risk-reducing effect among those with > 0– ≤ 1 drinks per day of alcohol may in part due to the sick-quitter effect, which assumes that continual non-drinkers may contain those with worsening health conditions, such as the presence of more comorbidities, and thereby result in higher incidence of health outcomes. This is in part supported in our results, which depict that compared to those who maintained non-drinking status, those who initiated alcohol intake had better health profiles such as fewer comorbidities (Supplementary Table 1). A reduced risk of mortality with a light increase in alcohol consumption was also observed among the older age population (≥ 60 years). Other alcohol-associated diseases, including injury, liver disease, and infectious disease occur earlier in the life course, and cardiovascular diseases occurred more frequently in the older population^22^. As a result, the influence of cardiovascular disease on mortality was more profound in the older population, and the protective effect of a light increase in alcohol consumption can be attributed to selection bias.

Determining the characteristics of the initial non-drinkers who start drinking is important in comprehending socioeconomic or behavioural factors that influence drinking, and it would be helpful to develop a strategy to maintain alcohol abstinence. A physically active lifestyle and risky behaviours are positively correlated^23^. Drinking was associated with an increased likelihood of engaging in exercise, and participants with moderate drinking performed exercise two-times more than did abstainers^24,25^. Extroverted personality, increased social anxiety, exercise motivated by guilt from unhealthy behaviours, alcohol drinking as a reward for intense physical activity, and coping mechanisms for exercise and drinking to avoid negative affect could be possible explanations for the positive association^23^. According to the current study, subjects who start drinking alcohol are more likely to be male, be physically active, have a higher socioeconomic status, be current smoker, and have less medical comorbidities. To prevent initiation of alcohol consumption, a tailored intervention based on understanding an individual’s motivation for drinking is necessary. Intervention to engage in other pleasure-related activities to substitute for alcohol use is appropriate for subjects who drink alcohol as a reward for hard work. Choosing customized, individualized exercise as a tool for enhancing social fun, enhancing affect, mitigating social anxiety, and subsequently reducing substance craving could also prevent initiation of alcohol use. Smoking cessation also needs to be combined with alcohol abstinence.

There are several limitations that need to be addressed. First, the current study utilizes data on alcohol consumption obtained using self-reported questionnaires. Self-reported alcohol consumption tends to be underreported, and sales data-based calculations may be more accurate in estimating the actual amount of alcohol consumption^26^. However self-reported measures of alcohol consumption were reasonably accurate among light-to-moderate drinkers and only heavy drinkers underestimated their consumption^27^. As less than 1% of the total subjects consumed more than 4 glasses of alcohol per day, the impact of underestimation by self-reporting may not alter the results of the current study. Second, a drinking habit, such as a pattern of episodic binge drinking, also has much influence on clinical outcome^28^. As there were limited number of subjects who episodically drank more than 4 glasses of alcohol per day among subjects with light-to-moderate alcohol consumption, a comparison between daily drinkers and episodic drinkers was not possible. Third, we could not exclude formal drinkers who had quit drinking because of multiple comorbidities even though the study population was derived from a health screening cohort and subjects with preexisting cardiovascular diseases were excluded. Finally, the study subjects consists of Korean population and generalizability to other countries and ethnicities may be limited. Future studies that determine the association of a change in alcohol consumption with the occurrence of MACE in populations with other ethnicities are needed.

In conclusion, a light increase in alcohol consumption to ≤ 1 glass per day from a non-drinker status significantly reduced the risk of MACE but did not reduce the risk of all-cause mortality. The beneficial effect of light alcohol increment on MACE may be attributed to selection bias and inappropriate inclusion of sick quitters in the reference group.

## Methods

Data collected by the National Health Insurance Service-Health Screening Cohort (NHIS-HEALS) between 2007 and 2013 were used for the analysis. NHIS-HEALS provides mandatory health insurance to all Korean citizens, resulting in an enrolment rate of 98%. Furthermore, the NHIS maintains databases of all insured health services, and a part of this data is provided for research purposes. The data are obtained from biennial general national health screening programs for early detection and primary prevention of diseases in Korean adults aged 40 years or older. The NHIS-HEALS database provides data on demographics (age, sex, income status), hospital utilization (admission date, discharge date, admission diagnosis, cost), and medical check-up results (body mass index, systolic and diastolic blood pressure, cholesterol profile, liver and renal function results, fasting blood glucose levels, family history of the disease, smoking habit, drinking habit, exercise status). The NHIS database has been validated and published elsewhere^29^.

Among the subjects who provided data on their drinking status at both the first (2007–2008) and second (2009–2010) medical check-ups, those who respond that they did not drink at all at the first NHIS medical check-up were included in the analysis. Participants who were diagnosed with cardiovascular disease (n = 43,632) or who died before the index date of 1 January 2011 (n = 255) and those with missing information on drinking habits at the second medical check-up (n = 3,187) or on age or sex (n = 1906) were excluded. The International Classification of Diseases, tenth version (ICD-10) codes and hospital admission data (≥ 2 days of admission) were used to identify stroke (I60-69), and coronary artery disease (I21-24) occurrence during the follow-up period from 1 January 2011 to 1 December 2013. Similarly, death due to external causes was defined as death caused by external circumstances including accidents, injury, assault, and poisoning (ICD-10 code: V01-Y98). All the participants were followed up starting from the index date of January 1, 2011, until the date of onset of a MACE death, or December 31, 2013, whichever came first.

Alcohol consumption, measured by glass per day at the second medical check-up (2009–2010), was categorized into maintenance of nondrinking (0), > 0– ≤ 1, > 1– ≤ 2, > 2– ≤ 4, and > 4 glasses per day. One glass of drink was considered to contain 10 g of ethanol regardless of beverage type. Hazard ratios (HRs), and 95% confidence intervals (CIs) for MACE, all-cause mortality according to the change in alcohol consumption were calculated using Cox regression analysis. Adjustment were made for conventional cardiovascular risk factors including age, sex, income, body mass index, exercise, history of hypertension, history of diabetes, systolic blood pressure, diastolic blood pressure, low density lipoprotein, high-density lipoprotein, and fasting blood glucose. The Charlson comorbidity index, which is one of the most widely used index of multiple comorbidities, was also used for risk adjustment. It is based on the ICD-10 codes from the database including 19 conditions, each of which is assigned a weight from 1 to 6 based on the predicted risk of mortality. The sum of each score predicts the 10-year survival of a person by weighting a number of comorbid conditions^30^. Stratified analysis of the association of alcohol consumption change was performed with MACE and all-cause mortality according to sex, age, physical activity, smoking, and Charlson Comorbidity Index (CCI).

Analyses were performed using SAS version 9.4 (SAS Institute Inc, Cary, NC). All tests were 2-sided, and a *p* value of ≤ 0.05 was considered significant.

This study was approved by the Seoul National University Institutional Review Board. The board waived the requirement for informed consent because of the retrospective nature of the study, and the NHIS-HEALS database was anonymized according to strict confidentiality guidelines before distribution. All methods were carried out in accordance with relevant guidelines and regulations.

## Supplementary information

Supplementary information.

## Data Availability

The data are available from the corresponding author upon reasonable request.
