# Supplementary material for: Association of change in alcohol consumption with cardiovascular disease and mortality among initial nondrinkers
Source: Sci Rep. 2020 Aug 7;10:13419. doi: 10.1038/s41598-020-70304-7 (PMC7414908; doi:10.1038/s41598-020-70304-7)

Association of Change in Alcohol Consumption with Cardiovascular Disease and Mortality Among Initial Nondrinkers

Jun Young Chang, MD[1*]; Seulggie Choi. MD[2*]; Sang Min Park, MD PhD MPH[2]

[1]Department of Neurology, Asan Medical Center, Seoul, Republic of Korea

[2]Department of Biomedical Sciences, Seoul National University Graduate School, Seoul Republic of Korea

Supplementary Table 1. Descriptive characteristics of the study population according to alcohol consumption initiation.

	Alcohol intake during the second medical checkup (drinks per day)			
	**Total**	**Maintained nondrinking status**	**Started drinking**	**P value**
Number of participants (%)	112,403	96,716 (86.0)	15687 (14.0)	
Age, years, mean (SD)	58.7 (8.5)	59.0 (8.6)	56.6 (7.7)	<0.01
Sex, n (%)				
Men	40,035 (35.6)	28,994 (30.0)	11041 (70.4)	<0.01
Women	72,368 (64.4)	67,722 (70.0)	4646 (29.6)	
Household income, quartiles, n (%)				<0.01
1st (highest)	37,640 (33.5)	31,672 (32.8)	5968 (38.0)	
2nd	32,879 (29.3)	28,277 (29.2)	4602 (29.3)	
3rd	24,482 (21.8)	21,356 (22.1)	3126 (19.9)	
4th (lowest)	17,402 (15.5)	15,411 (15.9)	1991 (12.7)	
Smoking, n (%)				
Never smoker	89,331 (79.5)	81,139 (83.9)	8192 (52.2)	<0.01
Past smoker	12,538 (11.2)	8,503 (8.8)	4035 (25.7)	
Current smoker	10,534 (9.4)	7,074 (7.3)	3460 (22.1)	
Physical activity, times per week, n (%)				<0.01
0	57,150 (50.8)	51,556 (53.3)	5594 (35.7)	
1 or 2	32,126 (28.6)	26,032 (26.9)	6094 (38.9)	
3 or 4	16,690 (14.9)	13,782 (14.3)	2908 (18.5)	
≥5	6,437 (5.7)	5,346 (5.5)	1091 (7.0)	
Body mass index, kg/m^2, mean (SD)	23.7 (2.9)	23.7 (2.9)	23.9 (2.8)	<0.01
Systolic blood pressure, mmHg, mean (SD)	123.3 (15.1)	123.2 (15.2)	124.1 (14.7)	<0.01
Fasting serum glucose, mg/dL, mean (SD)	98.3 (22.6)	98.0 (22.3)	100.3 (24.1)	<0.01
Total cholesterol, mg/dL, mean (SD)	202.5 (37.1)	202.8 (37.2)	200.4 (36.2)	<0.01
Charlson comorbidity index, n (%)				<0.01
0	24,261 (21.6)	20,096 (20.8)	4165 (26.6)	
1	33,927 (30.2)	28,954 (29.9)	4973 (31.7)	
2	26,300 (23.4)	22,840 (23.6)	3460 (22.1)	
≥3	27,915 (24.8)	24,826 (25.7)	3089 (19.7)	

Supplementary Figure 1. Flow diagram of the study subjects

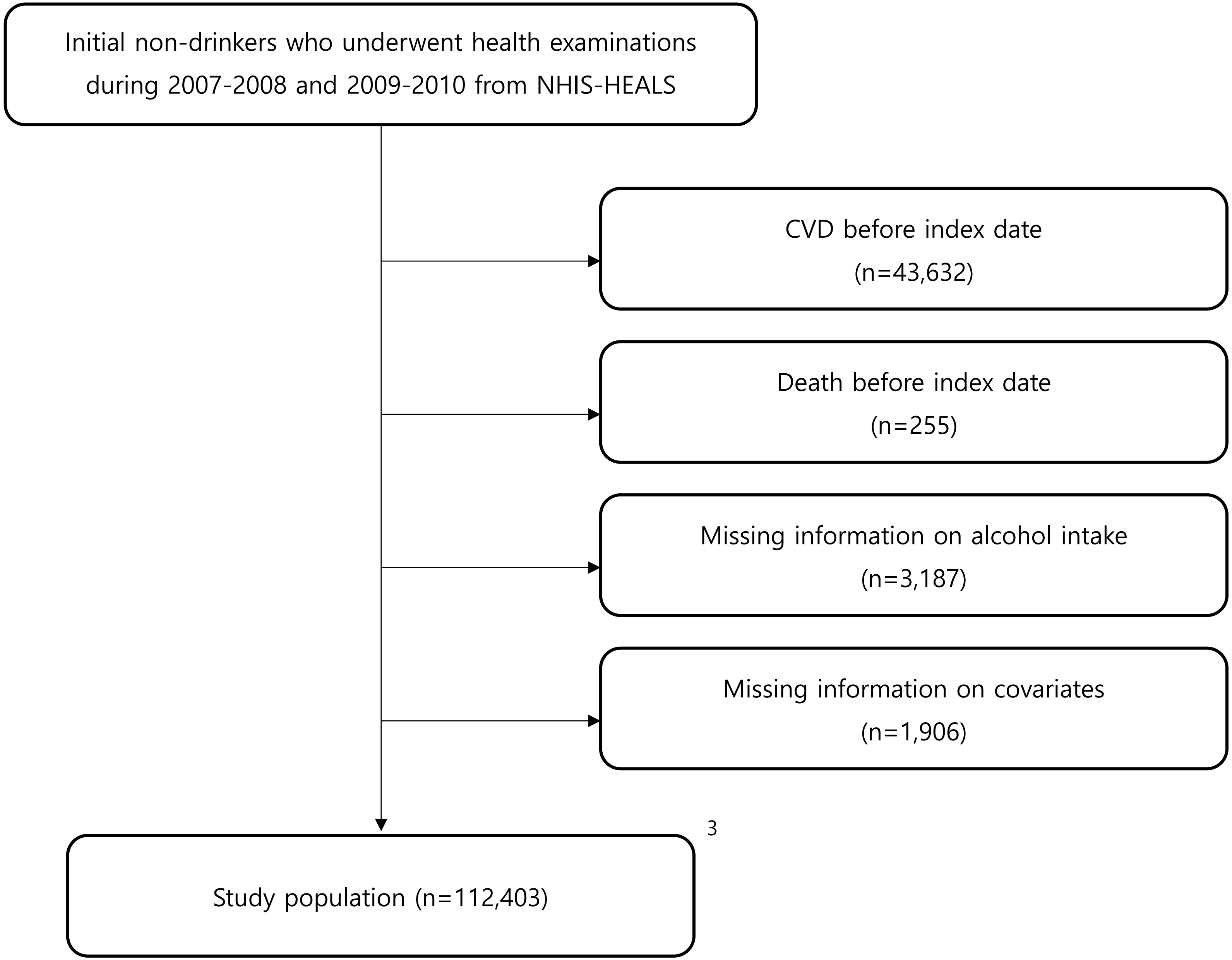

Supplement: Supplementary file 1 — Supplementary information. [file 41598_2020_70304_MOESM1_ESM.pdf]
